# The *Arabidopsis* glutamyl-tRNA reductase (GluTR) forms a ternary complex with FLU and GluTR-binding protein

**DOI:** 10.1038/srep19756

**Published:** 2016-01-22

**Authors:** Ying Fang, Shun Zhao, Feilong Zhang, Aiguo Zhao, Wenxia Zhang, Min Zhang, Lin Liu

**Affiliations:** 1 Key Laboratory of Photobiology, CAS Center for Excellence in Molecular Plant Sciences, Institute of Botany, Chinese Academy of Sciences, Beijing, 100093, China; 2 University of Chinese Academy of Sciences, Beijing, 100049, China; 3 School of Life Sciences, Anhui University, Hefei, Anhui, 230601, China; 4 College of Life Sciences, Northwest A&F University, Xi’an, Shaanxi, 712100, China

## Abstract

Tetrapyrrole biosynthesis is an essential and tightly regulated process, and glutamyl-tRNA reductase (GluTR) is a key target for multiple regulatory factors at the post-translational level. By binding to the thylakoid membrane protein FLUORESCENT (FLU) or the soluble stromal GluTR-binding protein (GBP), the activity of GluTR is down- or up-regulated. Here, we reconstructed a ternary complex composed of the C-terminal tetratricopepetide-repeat domain of FLU, GBP, and GluTR, crystallized and solved the structure of the complex at 3.2 Å. The overall structure resembles the shape of merged two binary complexes as previously reported, and shows a large conformational change within GluTR. We also demonstrated that GluTR binds tightly with GBP but does not bind to GSAM under the same condition. These findings allow us to suggest a biological role of the ternary complex for the regulation of plant GluTR.

Plants synthesize δ-aminolevulenic acid (ALA), the precursor for all tetrapyrrole molecules, from glutamate via a three-step pathway^1^. The first step is ligation of glutamate to tRNA^Glu^ catalyzed by glutamyl-tRNA synthetase. Then glutamyl-tRNA reductase (GluTR) reduces the tRNA^Glu^-bound glutamate to glutamate-l-semialdehyde (GSA) in an NADPH-dependent manner. GSA is subsequently isomerized to ALA by a vitamin B_6_-dependent enzyme, glutamate-l-semialdehyde aminomutase (GSAM). ALA synthesis is the key regulatory point for the entire tetrapyrrole biosynthetic pathway, and particularly GluTR is subjected to a tight control at the post-translational level^2^,^3^.

Three mechanisms have been characterized for plant GluTR activity regulation, which are (i) the end-product feedback inhibition by heme^4^, (ii) repression by a membrane protein FLUORESCENT (FLU)^5^, and (iii) formation of complex with a soluble GluTR-binding protein (GBP)^6^. The two inhibitors, heme and FLU, are suggested to concurrently interact with different sites on GluTR^7^. GluTR consists of three domains: an N-terminal catalytic domain, an NADPH-binding domain, and a C-terminal dimerization domain^8^. FLU directly interacts with GluTR’s dimerization domain through its tetratricopepetide-repeat (TPR) domain^7^,^9^,^10^. Plant GluTRs have an ~30-residue conserved fragment in the N-terminal region, and truncation of this fragment results in resistance to heme inhibition^4^. This putative heme-binding fragment, however, is flexible and hence not observed in the GluTR-GBP complex structure^11^. GBP has been proposed to protect GluTR from FLU inhibition during darkness to ensure heme synthesis when the need for chlorophyll declines^12^, and a membrane anchoring protein specific for GBP has been speculated^13^. Recent structural studies of the GluTR-GBP complex^11^ and of FLU’s TPR domain (FLU^TPR^) complexed with GluTR’s dimerization domain^10^ have revealed that FLU and GBP bind to different sites on GluTR. These findings indicate that the three post-translational mechanisms of GluTR regulation may function simultaneously.

Transcriptional regulation of enzymes involved in ALA synthesis has been characterized in *Arabidopsis thaliana*. Among the three GluTR genes (*HEMA1*, *HEMA2* and *HEMA3*), expression of *HEMA1* that encodes the dominant GluTR in the photosynthetic tissues is regulated by light^14^,^15^,^16^. Light also regulates expression of the genes encoding GSAM^14^ and ALA dehydratase, the enzyme subsequent to GSAM in the tetrapyrrole biosynthetic pathway^17^. Expression of FLU and GBP, however, is not sensitive to light change^6^,^7^. The loss-of-function mutation of either *flu* or *gbp* is lethal^5^,^6^, highlighting a critical role for these two constitutively expressed proteins.

Aside from FLU and GBP, GSAM is proposed to form complex with GluTR to enable GSA channeling from GluTR to GSAM^8^. The GluTR-GSAM complex has been observed for these two enzymes from *Escherichia coli*^18^ and from the unicellular green alga *Chlamydomonas reinhardtii*^19^. However, in plants, there is no biological evidence for the GluTR-GSAM complex formation. Enzymes after ALA synthesis and before the heme-chlorophyll branch point (protoporphyrin IX) are speculated to physically interact to form transient substrate-channeling complexes^13^,^20^. It is therefore worthy to test whether a stable plant GluTR-GSAM complex exists by using an *in vitro* system.

GluTR and its three partner proteins, FLU, GBP and GSAM, are homodimers. The 2:2 FLU^TPR^-GluTR complex and the 2:2 GluTR-GBP complex have been reconstructed^10^,^11^. In the present study, we obtained the 2:2:2 FLU^TPR^-GluTR-GBP complex and solved its structure. We show that GBP has higher affinity to GluTR than FLU^TPR^ when quantified by isothermal titration calorimetry (ITC) experiment. ITC did not detect GSAM binding to GluTR or to the GluTR-GBP complex. These results advance the understanding of plant GluTR regulation at the molecular level and provide a clue to the spatial organization of these proteins.

## Results

### Reconstruction, crystallization and structure determination of the ternary complex

**Figure 1:**
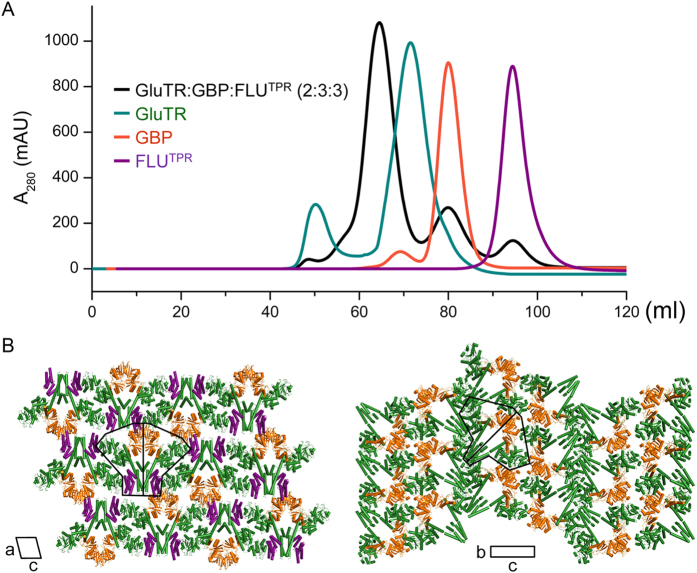
Reconstruction and crystallization of the FLU^TPR^-GluTR-GBP ternary complex. (**A**) Elution profiles of FLU^TPR^, GluTR, GBP, and their mixture. Y-axis: mAU, milli-absorbance units; x-axis: volume in ml. (**B**) Crystal packing of the ternary complex (*left panel*) and its comparison with the GluTR-GBP complex (*right panel*). Color scheme: FLU^TPR^, purple; GluTR, green; GBP, orange. The outline and the local 2-fold axis of a protein complex are in black lines. The ternary complex (*left panel*) is viewed from a direction perpendicular to the crystallographic a–c plane; the GluTR-GBP complex (*right panel*), to the b,c plane.

The purified recombinant GluTR, GBP and FLU^TPR^ were mixed at molar ratio of 2:3:3, and the mixture was then subjected to size-exclusion chromatography. A stable FLU^TPR^-GluTR-GBP ternary complex was obtained with excess amounts of GBP and FLU^TPR^ (Fig. 1A). No complex formation between FLU^TPR^ and GBP was observed. Fractions corresponding to the ternary complex were concentrated for crystallization. Crystals grew under a totally different condition from the GluTR-GBP complex^11^ or FLU^TPR^ in complex with GluTR’s dimerization domain (GluTR^DD^)^10^. The ternary complex crystals belong to space group *C*2, while the GluTR-GBP binary complex crystals belong to *P*2_1_2_1_2_1_, and the FLU^TPR^-GluTR^DD^ binary complex crystals belong to *P*6_5_22. The ternary complex packs in a symmetric way along its local 2-fold axis, whereas the GluTR-GBP complex arrays in an asymmetric way along the axis (Fig. 1B). The structure of the ternary complex was determined by the molecular replacement method using template coordinates of GluTR-GBP and FLU^TPR^-GluTR^DD^, and refined to a resolution of 3.2 Å (Table 1).

### Structure of the ternary complex

**Figure 2:**
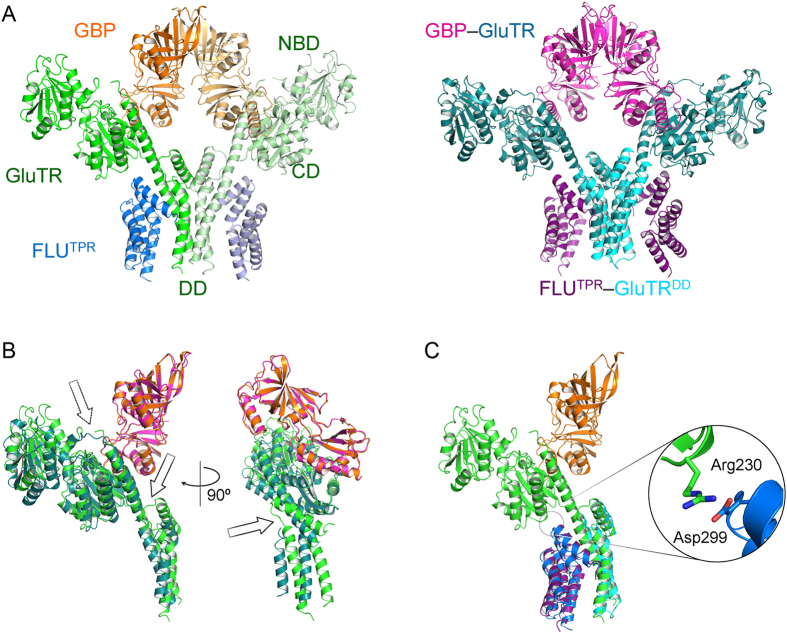
Structure of the FLU^TPR^-GluTR-GBP ternary complex. (**A**) Overall structure of the ternary complex and a superimposition of the two binary complexes. For the ternary complex (*left panel*), FLU^TPR^, GluTR and GBP are colored coded as per each monomer. The catalytic domain (CD), NADPH-binding domain (NBD) and dimerization domain (DD) of GluTR are indicated on one GluTR monomer. For the two binary complexes (*right panel*), FLU^TPR^, GluTR^DD^, GluTR and GBP are colored coded as per each dimer. (**B**) Structural comparison of the GluTR-GBP part in the ternary complex with the GluTR-GBP complex. Structures are colored as in (**A**), and only halves are shown. The dashed arrow indicates the missing region in the ternary complex; the solid arrow indicates the region observed in the ternary complex but not in the GluTR-GBP complex. The structures are rotated 90 degrees along the y-axis to show the difference in the dimerization domain of GluTR. (**C**) Structural comparison of the ternary complex with the FLU^TPR^-GluTR^DD^ complex. The inset shows the details of the ionic bond between the catalytic domain of GluTR and FLU^TPR^. The abbreviations used are as follows: CD, the catalytic domain of GluTR; NBD, the NADPH-binding domain of GluTR; DD, the dimerization domain of GluTR; FLU^TPR^, the recombinant TPR domain of FLU; GluTR^DD^, the recombinant dimerization domain of GluTR.

The ternary complex resembles the shape of a merged structure of the two binary complexes (Fig. 2A). However, it does not fit well with either GluTR-GBP or FLU^TPR^-GluTR^DD^. The positions of GluTR’s C-terminal region are quite different when the ternary complex and GluTR-GBP are superimposed (Fig. 2B). GBP and the remainder of GluTR have no significant change, except that the linker between NADPH-binding domain and the long “spinal” helix of GluTR cannot be traced in the ternary complex. Conversely, the linker between the two C-terminal helices of GluTR that is missing in GluTR-GBP or FLU^TPR^-GluTR^DD^ can be observed in the ternary complex. Compared with FLU^TPR^-GluTR^DD^, there is an extra ionic bond between the catalytic domain of GluTR and the third TPR motif of FLU^TPR^ (Fig. 2C). GluTR’s C-terminal region appears more compact in the ternary complex than in FLU^TPR^-GluTR^DD^.

### Flexibility of GluTR’s spinal helix

**Figure 3:**
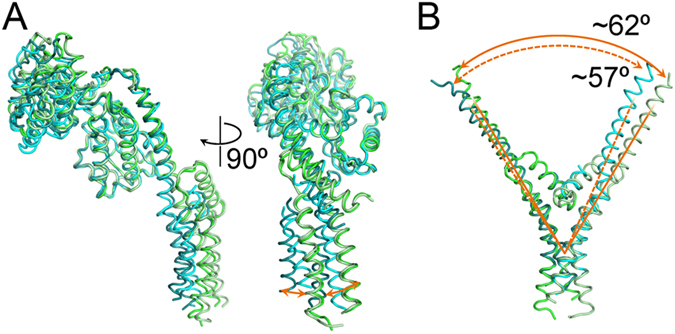
Superimposition of GluTR monomers. (**A**) Superimposed backbones of the four GluTR monomers. The color schemes are green for chain (**A**) of 5CHE, light green for chain (**B**) of 5CHE, prussian blue for chain (**A**) of 4N7R, and cyan for chain (**B**) of 4N7R. Orange arrows denote structural difference in the dimerization domain. (**B**) Superimposed backbones of the spinal helix and the dimerization domain of the two GluTR dimers. The two arms of the Y-shaped GluTR dimer in the ternary complex are denoted by solid orange lines; the two arms in the GluTR-GBP binary complex are denoted by dashed orange lines.

The two chains of GluTR in the ternary complex, together with the previous observation in the GluTR-GBP complex^11^, demonstrate the flexibility of GluTR’s spinal helix across a large range. When the catalytic domains of the four chains are superimposed, the stem end exhibits maximum shift of approximate 15 Å (Fig. 3A). The two spinal helices in the ternary complex are almost identical, which is reminiscent of *Methanopyrus kandleri* GluTR^8^. Indeed, the root-mean-square deviation between the two chains of GluTR in the ternary complex is only 0.72 Å for 419 Cα aligned. When the stem of the GluTR dimer in the GluTR-GBP binary complex is superimposed with that in the ternary complex, the angle between the two Y-shaped arms has a difference of ~5 degrees (Fig. 3B).

### GluTR’s interaction with GSAM and GBP

**Figure 4:**
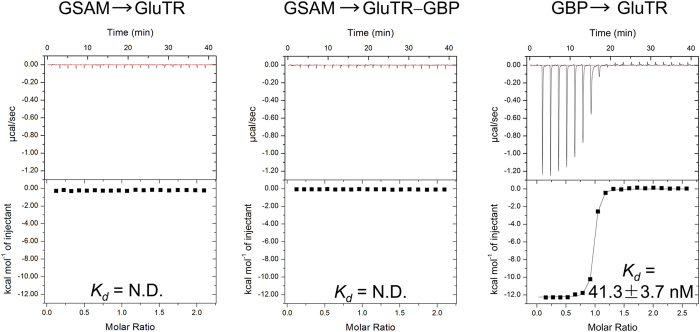
ITC analysis of GluTR’s interaction with GSAM and GBP. The *top panel* shows the heat response upon each injection, and the *bottom panel* shows the integrated heat value (▪) and the fit (−) to a single-site binding model. N.D., not determined.

GSAM is a flexible enzyme undergoing open/close conformational change^21^,^22^. Synchronized events between GluTR and GSAM are likely required for GluTR-GSAM interaction. A stable GluTR-GSAM complex has been verified for this pair of enzymes from *E. coli* and *C. reinhardtii*^18^,^19^. In contrast, direct interaction between plant GluTR and GSAM has not been reported. We employed ITC to detect such interaction (Fig. 4). No heat change was observed for titration of GSAM to GluTR. Also, no heat change was observed for titration of GSAM to the GluTR-GBP complex. Notably, the GluTR-GBP complex is stable and has a low apparent dissociation constant (*K*_*d*_). The *K*_*d*_ value (41.3 ± 3.7 nM) is about one-fortieth that of FLU^TPR^ and GluTR as measured previously^10^, which indicates that GBP binds significantly more tightly than FLU^TPR^ to GluTR.

## Discussion

As the rate-limiting step for the formation of ALA, the common precursor for all tetrapyrrole molecules, the GluTR-catalyzed glutamyl-tRNA^Glu^ reduction by NADPH is a key regulatory point of the tetrapyrrole biosynthetic pathway^2^,^3^. The membrane-anchored protein FLU were identified as a negative regulator for GluTR^5^,^7^,^9^. The soluble protein GBP was initially found in chloroplast stroma^23^, and then later in a thylakoid membrane-bound 300-kDa protein complex^6^. Direct GluTR-GBP interaction has also been found in an interactome screen^24^. With the components of the 300-kDa protein complex remaining unresolved, the FLU^TPR^-GluTR-GBP ternary complex presented here provides a clue to address this issue. A membrane-bound FLU-containing metabolic complex has been detected by an immunoprecipitation/mass spectrometry study^25^. In this complex, light-dependent protochlorophyllide oxidoreductase (LPOR) is one of the specific FLU-interacting partners. LPOR catalyzes the reduction of the fourth ring of protochlorophyllide, and exists as dimers or tetramers^26^. With a monomeric molecular weight of ~36 kDa, when a LPOR dimer binds to the ternary complex of full-length FLU-GluTR-GBP that has a combined molecular weight of ~224 kDa, the resulting LPOR-FLU-GluTR-GBP quaternary complex might explain the post-translational regulation of ALA synthesis by light^6^,^27^. Further biological studies are needed to characterize such a macromolecular assembly.

How GBP exerts its regulatory role on GluTR activity remains an open question. GBP has higher binding affinity to GluTR compared with FLU^TPR^. This indicates that GluTR is more prone to bind to GBP than FLU under the same condition. GBP may regulate GluTR activity by the following three mechanisms: (i) to shift GluTR conformation and render GluTR preferable for NADPH accommodation within the NADPH-binding domain, and thus prevent GluTR’s esterase activity; (ii) to retain GSA in GluTR’s interior before GSAM interaction; (iii) to be involved in chloroplast vitamin B_6_ metabolism and hence related to GSAM activation.

The failure to detect GluTR-GSAM interaction using ITC does not preclude the existence of a GluTR-GSAM complex in plants. Nevertheless, such a complex might be less stable than its counterparts from *E. coli* and *C. reinhardtii*^18^,^19^. It should be noticed that, similar to a GluTR dimer, a GSAM dimer has both asymmetric and symmetric states^21^,^22^,^28^. A synchronized conformational change of both GluTR and GSAM is likely required for their recognition. In addition, whether and how dissociation of the GluTR-GBP complex is involved in GSAM interaction remains unclear and awaits future biochemical characterization.

## Methods

### Protein expression and purification

The expression vectors of *Arabidopsis* GluTR, GBP and FLU^TPR^ were constructed as previously described^10^,^11^. Briefly, the genes of GluTR (At1g58290) and GBP (At3g21200) without their chloroplast localization sequences (residues 73–543 and 42–317), and the FLU (At3g14110) truncation (residues 195–316), were constructed into expression vectors pMAL-c5X (New England Biolabs), pET-28a(+) and pET-22b(+) (Novagen), respectively. The mature *Arabidopsis* GSAM (At3g48730), starting at residue 38, was cloned into the pETMALc-H vector^29^ between *Bam*HI and *Not*I sites. The constructs were transferred into *E. coli* BL21(DE3) cells, and protein expression was induced by 0.2 mM isopropyl β-D-1-thiogalactopyranoside when the cell density reached an optical density at 600 nm of 0.8. The induced cells were grown at 18 ^o^C for 16 hours before harvest by centrifugation.

The cell pellets expressing GluTR were re-suspended in buffer A (20 mM Tris-HCl, pH 7.5, 500 mM NaCl, 1 mM EDTA and 5 mM dithiothreitol) and disrupted by sonication. After centrifugation, the cleared lysate was passed through a maltose binding protein (MBP) affinity column pre-equilibrated with buffer A. The bound protein was eluted with 40 mM maltose in buffer A, and the MBP tag was then cleaved using tobacco etch virus protease. The reaction mixture was then subjected to a HiLoad 16/60 Superdex 200 pg column (GE Healthcare) pre-equilibrated and eluted with buffer A. Protein aggregates and the MBP tag were removed, and the GluTR dimer fractions were collected. Purification of GBP and FLU^TPR^ was described previously^10^,^11^. GSAM was purified following the same procedure used for GluTR as described above.

### Reconstruction of the ternary complex

For preparation of the ternary complex, the GluTR dimer fractions were mixed with GBP and FLU^TPR^ at a molar ratio 2:3:3 and incubated for 1 hour at 4 ^o^C. The mixture was loaded on a HiLoad 16/60 Superdex 200 pg column (GE Healthcare) pre-equilibrated and eluted with buffer containing 20 mM Tris-HCl, pH 7.5, 200 mM NaCl, and 4 mM dithiothreitol. The purified ternary complex was pooled and concentrated to 15 mg ml^−1^ for crystallization.

### Crystallization and data collection

Crystals of the ternary complex were obtained in 0.1 M sodium malonate, pH 7.0, 14.5% (w/v) polyethylene glycol 3,350, 2.5% (v/v) 2-methyl-2,4-pentanediol, 0.5 M lithium chloride by the sitting-drop vapor diffusion method at 16 ^o^C. For data collection, the crystals were transferred step by step into drops of the crystallization liquid supplemented with 5%, 10%, 20% (v/v) ethylene glycol before being flash-frozen in liquid nitrogen. All X-ray diffraction data sets were collected at beamline BL17U of Shanghai Synchrotron Radiation Facility (Shanghai, China) at 100 K, and processed using HKL2000 (HKL Research, Inc.).

### Structure solution and refinement

The structural model of the ternary complex was built using molecular replacement with Phaser^30^. The search templates were the GluTR-GBP complex (PDB entry 4N7R) where residues after Arg421 of GluTR were removed, and FLU^TPR^-GluTR^DD^ (PDB entry 4YVQ). Manual correction was done in Coot^31^ according to the *2F*_*o*_ − *F*_*c*_ and *F*_*o*_ − *F*_*c*_ electron density maps. Further refinement was performed with phenix.refine^32^. The diffraction data used for structure refinement was extended to 3.0 Å according to CC_1/2_ values^33^, and the final resolution was cut off to 3.2 Å based on traditional restriction. The overall quality of final structure was assessed by MolProbity^34^ with 96.7% in favored, 3.0% in general allowed and 0.3% in disallowed regions. Data collection and structure refinement statistics are summarized in Table 1. The protein structure figures were prepared with the program PyMOL (Schrödinger, LLC).

### Isothermal titration calorimetry

ITC experiments were performed on a MicroCal iTC200 calorimeter (Malvern Instruments Ltd) at 25 ^o^C. The purified recombinant proteins were changed into a buffer containing 20 mM Tris-HCl, pH 7.5, and 150 mM NaCl. Each titration experiment consisted of 20 injections of 2 μl aliquots of the protein at a concentration of 0.5 mM into a 200 μl cell filled with protein at a concentration of 50 μM. Control experiments were carried out by injecting protein into the buffer, and the resulting heat of dilution was subtracted. The first injection was discarded, and the data were fitted to a one-site binding model using MicroCal Origin software.

## Additional Information

**Accession codes:** Atomic coordinates and structure factors have been deposited with the Protein Data Bank under accession code 5CHE.

**How to cite this article**: Fang, Y. *et al*. The *Arabidopsis* glutamyl-tRNA reductase (GluTR) forms a ternary complex with FLU and GluTR-binding protein. *Sci. Rep.* **6**, 19756; doi: 10.1038/srep19756 (2016).

## Figures and Tables

**Table 1:** Crystallographic data and refinement statistics.

PDB code	5CHE
Data collection	
Space group	C2
Unit cell dimensions	
a, b, c (Å)	217.0, 53.2, 203.8
α, β, γ (°)	90.0, 108.4, 90.0
Wavelength (Å)	0.9793
Resolution (Å)	50.0–3.20 (3.31–3.20)
No. of measured reflections	156901
No. of unique reflections	36775 (3661)
Completeness (%)	99.6 (99.9)
Redundancy	4.3 (4.3)
I/σI	10.0 (1.7)
R_merge_	0.132 (0.831)
Refinement statistics	
Resolution (Å)	39.27–3.20 (3.30–3.20)
R_work_/R_free_(%)	22.3/27.8
Number of atoms	
Protein	12365
Water	6
Average B value (Å^2^)	25.78
R.m.s deviations	
Bond lengths (Å)	0.006
Bond angles (°)	0.975
Ramachandran plot	
Most favored (%)	96.67
Additional allowed (%)	3.01
Disallowed (%)	0.32

*R*_*merge*_ = Σ_hkl_Σ_i_|*I*_*hkl,i*_ − *I*_*m*_|/Σ_hkl_Σ_i_*I*_*hkl,i*_, where *I*_*hkl,i*_ is the intensity of the measured reflection and *I*_*m*_ is the mean intensity of the symmetry-related reflections after rejections. R = Σ||*F*_*o*_| − |*F*_*c*_||/Σ*|F*_*o*_*|,* where *F*_*o*_ and *F*_*c*_ are the observed and calculated structure factors, respectively. R_free_ is the cross-validated R-factor computed for a test set of 5% of the reflections, which were omitted during refinement. The values in parentheses relate to the highest resolution shell.
